# Radiation shielding evaluation based on five years of data from a busy CyberKnife center

**DOI:** 10.1120/jacmp.v15i6.4575

**Published:** 2014-11-08

**Authors:** Jun Yang, Jing Feng

**Affiliations:** ^1^ Philadelphia CyberKnife Havertown PA; ^2^ Radiation Oncology, Drexel University Philadelphia PA; ^3^ Medical Physics, Alliance Oncology Nashville TN USA

**Keywords:** CyberKnife, radiation shielding, guidelines, use factor

## Abstract

We examined the adequacy of existing shielding guidelines using five‐year clinical data from a busy CyberKnife center. From June 2006 through July 2011, 1,370 patients were treated with a total of 4,900 fractions and 680,691 radiation beams using a G4 CyberKnife. Prescription dose and total monitor units (MU) were analyzed to estimate the shielding workload and modulation factor. In addition, based on the beam's radiation source position, targeting position, MU, and beam collimator size, the MATLAB program was used to project each beam toward the shielding barrier. The summation of the projections evaluates the distribution of the shielding load. On average, each patient received 3.6 fractions, with an average 9.1 Gy per fraction prescribed at the 71.1% isodose line, using 133.7 beams and 6,200 MU. Intracranial patients received an average of 2.7 fractions, with 8.6 Gy per fraction prescribed at the 71.4% isodose line, using 133 beams and 5,083 MU. Extracranial patients received an average of 3.94 fractions, with 9.2 Gy per fraction prescribed at the 71% isodose line, using 134 beams and 6,514 MU. Most‐used collimator sizes for intracranial patients were smaller (7.5 to 20 mm) than for extracranial patients (20 to 40 mm). Eighty‐five percent of the beams exited through the floor, and about 40% of the surrounding wall area received no direct beam. For the rest of the wall, we found “hot” areas that received above‐average MU. The locations of these areas were correlated with the projection of the nodes for extracranial treatments. In comparison, the beam projections on the wall were more spread for intracranial treatments. The maximum MU any area received from intracranial treatment was less than 0.25% of total MU used for intracranial treatments, and was less than 1.2% of total MU used for extracranial treatments. The combination of workload, modulation factor, and use factor in our practice are about tenfold less than recommendations in the existing CyberKnife shielding guidelines. The current guidelines were found to be adequate for shielding, even in a busy center. There may be a potential to reduce shielding in areas with no or few direct beams in the current G4 model. Since a newer model CyberKnife (M6) has recently been introduced, the patterns of usage reported here may be changed in the future. The uneven distribution of use factor we have found may, however, be considered in the vault design.

PACS number: 87.55.N

## I. INTRODUCTION

Unlike gantry‐based linear accelerators (conventional linacs), which deliver radiation within a single plane through the isocenter, the CyberKnife delivers many 6 MV noncoplanar pencil beams from a wide solid angle around the target. Every beam originates from one of 120 preconfigured positions, or “treatment nodes”, that are nearly evenly distributed around the imaging alignment center. The centroid of a treatment target is located close to the alignment center, but not necessarily right on the alignment center. More than one beam can be directed from a single node (aiming at different portions of the target), and some nodes may not have an active beam depending upon planning optimization. Because of this configuration, CyberKnife beams are distributed to a very large part of the surrounding walls. As a result, CyberKnife vaults require a much larger primary shielding barrier than conventional linacs, but the use factor is much smaller. Another difference from conventional linac treatments is that the CyberKnife is used almost exclusively for radiosurgery and stereotactic body radiotherapy (SBRT), so a very high per‐fraction dose is delivered in relatively few fractions per treatment course.

These different characteristics have been addressed in the CyberKnife shielding guidelines. Three of these guidelines are widely accepted by CyberKnife physicists: NCRP 151 has been the gold standard guideline for shielding design.^(1)^ A book chapter published in 2005,^(2)^ “CyberKnife Treatment Room Design & Radiation Protection”, was the first published guideline and defined the shielding concepts that still stand today. These two guidelines estimated the primary workload and use factor based on the experience of the Georgetown University CyberKnife program.^(3)^ The shielding white paper^(4)^ from Accuray Incorporated, manufacturer of the CyberKnife, is valuable in addressing leakage dose and secondary barrier requirements, based on multiple centers' clinical data on modulation factor. All three of these guidelines propose that all surrounding walls serve as the primary shielding barrier with 0.05 as a uniform use factor for each wall.

We examined the adequacy of these shielding guidelines using five years of clinical data from a busy CyberKnife program. We report our workload and shielding‐related clinical characteristics, and provide additional suggestions based on our finding of an uneven distribution of use factor across the vault floor and walls.

## II. MATERIALS AND METHODS

### A. Data collection

We treated 1,370 cases, including 29% (392) intracranial cases and 71% (978) extracranial cases, with a total of 4,900 fractions at Philadelphia CyberKnife from June 2006 through July 2011. The CyberKnife model was G4 with the software version 7.x, upgraded to version 8.x in February 2009. Every patient was treated using one or two cone collimators selected from 12 circular field sizes: 5, 7.5, 10, 12.5, 15, 20, 25, 30, 35, 40, 50, and 60 mm in diameter. A one‐head path with 120 nodes was used to treat all brain patients, except for 11 trigeminal neuralgia patients treated with a special trigeminal path. The one‐body path has around 90 nodes, which excludes the nodes distributed on the apex of the skull due to patient safety concerns and the mechanical limitation of robot. The one‐body path was adopted to treat all extracranial patients, except for the prostate patients who were treated using a special prostate path.

**Figure 1:**
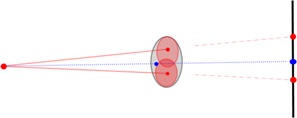
The illustration of beam projection and node projection. Assume there are two beams (with central axis marked as solid red line) from one treatment node (red solid dot on left), each irradiating a different portion of the target (in gray) and the two beams partial overlap with each other. The beams are projected to the wall on the right and the node projected through the alignment center (solid blue dot in the tumor) to the different location of the wall. Depending on the planning and patient anatomy, the alignment center can be located within the tumor, or outside the tumor.

Clinical data including prescription dose, fractionation schemes and MUs were retrospectively analyzed to estimate the workload and modulation factor. The 680,691 delivered radiation beams were simulated using MATLAB software (MathWorks, Natick, MA) to calculate the distribution of shielding load and to estimate the use factor. Based on the geometric positions of each beam's radiation source and its specific targeting position, each beam was projected to the floor or the surrounding barrier wall, and weighted by monitor units (MU) and collimator size (Fig. 1). In contrast to conventional linac technology where beams from the same gantry angle go through the same isocenter and project to the same directions, the CyberKnife beams from one node do not have same targeting positions and do not project to the same directions for the two following facts: 1) within one CyberKnife plan, the beams from a node irradiate different potions of the tumor with a wide range of overlap with each other (0% to about 90%), therefore do not project to the same destination; 2) more effectively, the tumors of different patients are at different locations relative to the alignment center, and the beams from same node irradiating the different tumors will project to different directions.

**Figure 2:**
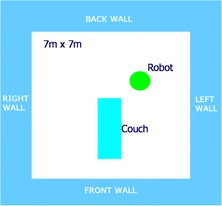
The layout of a hypothetical square CyberKnife vault with four surrounding walls, marked as Front, Right, Back and Left wall.

The accumulation of the projected MUs can be used to evaluate the distribution of the shielding load in a hypothetical vault with the inner space of 7m×7m×3m. (Fig. 2). In most cases the CyberKnife robot is positioned on the left superior side of couch, but in our center the robot is on the right superior side of the couch (known as the mirrored position). The distance from the radiation source to the entry point of a shielding wall is not factored in the accumulation in order to estimate the use factor appropriately. To simplify the calculation, “perfect” beams with 100% uniform dose in the field and 0% dose outside the field (as distinct from the real beam and its penumbra), were used.

### B. Shielding guidelines

Because CyberKnife's primary beams exit onto a large portion of the surrounding walls and the robot can be moved to a different location in the new model (M6), all three shielding guidelines recommend designing the surrounding walls as primary barriers and designing the roof or ceiling as a secondary barrier since the CyberKnife is limited in its ability to point in an upward direction.

Based primarily on the Georgetown experience, which found the largest number of beam MUs originating from one node is about 5% of total number of MUs in a single plan, a use factor of 5% is generally recommended in designing wall shielding. Georgetown's experience also found an average modulation factor of 15 for both intracranial and extracranial cases. A survey of multiple CyberKnife centers, cited in Accuray's shielding white paper,^(4)^ found an average modulation factor of 12.5 (range from 9.2 to 25) and also recommended a modulation factor of 15 as a reasonable conservative recommendation. Since the field sizes from CyberKnife are relatively small when compared with those from a conventional linac fields, the guidelines suggest that the amount of radiation workload generated from patient scatter is negligible.

We summarize the guidelines recommended parameters in Table 1, along with our clinical data.

**Table 1:** Summary of recommended parameters in the CyberKnife shielding design guidelines and the parameters found from the clinical data of Philadelphia CyberKnife.

	*Guidelines Recommendation*			
	*NCRP 151*	*White Paper* ^*(4)*^	*Book Chapter* ^*(2*^	*Our Clinical Data*
Max Num. of Tx per Day	8	8	6	9
Average Dose Per Tx (Gy)	12.5	12.5	12.5	9.1
Workload (Gy/week at 80 cm SAD)	500	500	375	400.4
MU per Tx		16,000		6,200
Modulation Factor	15	15	15	7.43
Use Factor	0.05	0.05	0.05	0.01

## III. RESULTS

### A. Primary shielding load

On average, patients received 3.6 fractions (SD 1.48), with a mean 9.1 Gy (SD 8.67) per fraction prescribed to the 71.1% (SD 7.02%) isodose line. Most frequently used fractionation schemes were 1 (14%), 3 (33.8%), and 5 (41.2%) fractions. Specifically, the intracranial patients received an average of 2.7 fractions (SD 1.74), with 8.6 Gy (SD 3.72) per fraction prescribed at the 71.4% (SD 7.69%) isodose line. Thirty‐two‐and‐a‐half percent (32.5%), 22.8% and 37% of intracranial patients received 1, 3, and 5 fractions, respectively, and a few intracranial patients received 2 or 4 fractions. The extracranial patients received an average of 3.94 fractions (SD 1.06), with 9.2 Gy (SD 3.63) per fraction prescribed at 71% (SD 6.65%) isodose line. Of the extracranical patients, 40.1%, 8.7%, and 46% received 3, 4, and 5 fractions, respectively, and only a few extracranial patients received 1 or 2 fractions.

**Figure 3:**
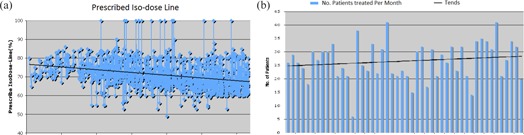
The history of prescribed isodose line (a) and the number of patients treated per month (b).

There have been a couple of studies supporting faster dose falloff when performing SBRT with higher dose heterogeneity.^5^, ^6^ In our practice, the average prescription isodose has dropped gradually, from the high 70% range to the high 60% range over the course of five years (Fig. 3(a)). This corresponds to a trend in our clinical practice toward maximizing dose falloff in surrounding tissue, and allowing greater heterogeneity within treatment volumes. An exception exists for prostate patients where we maintained the prescribed isodose line at around 83%.

On average, 27 patients (SD 7) were treated with 95 fractions (SD 25) every month. During the highest‐volume year, 347 patients were treated with 1,203 fractions. In the highest‐volume month, 41 patients were treated with 145 fractions. During the highest‐volume week, 44 fractions were delivered with a total of 381 Gy. The history of our monthly patient treatment volume is reported in Fig. 3(b).

The distribution of collimator use for 5, 7.5, 10, 12.5, 15, 20, 25, 30, 35, 40, 50, and 60 mm collimators are 1.1%, 4.3%, 5.3%, 5.5%, 8.5%, 14.8%, 11.2%, 13.4, 14.9%, 12.7%, and 5.6%. Most commonly used collimator sizes for intracranial patients were usually smaller (7.5, 10, 12.5, 15, and 20 mm) than those used for extracranial patients (20, 25, 30, 35, and 40 mm).

### B. Leakage load and modulation factor

On average, intracranial patients received 8.61 Gy (SD 3.72) per fraction with 5,083 MUs (SD 2268) and 133 (SD 50) beams, which resulted in an average of modulation factor of 6.2 (SD of 2.99) for intracranial patients. A mean of 38.2 MUs per beam per fraction were used in intracranial treatments. With an average 6517 (SD 3040) MU delivered to extracranial patients, who received 9.2 Gy (SD 3.63) per fraction in 134 (SD 49) beams, the modulation factor for extracranial patients was 7.93 (SD 4.09), higher than intracranial patients due to greater tissue attenuation. A mean of 53.8 MUs per beam per fraction was delivered in these extracranial treatments. The weighted average modulation factor was 7.43 (SD 3.79), which is about 50% of the recommended value or the multisite survey value seen in the published guidelines. As noted, our practice has been emphasizing dose falloff in the surrounding tissue, and conformality index is not our first planning optimization goal, since a plan with higher conformality often has a slower dose falloff and may require more MUs.^(6)^

### C. Shielding load distribution and use factor

#### C.1 Intracranial patient

**Figure 4:**
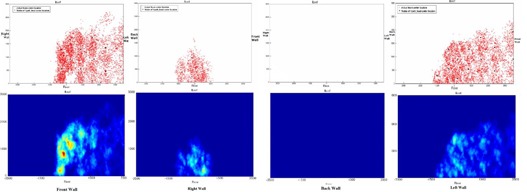
The projection of active radiation beams (red dot) and nodes (black dot) used in intracranial cases, and the corresponding MU weighted shielding load distribution. There is no active beam projected to the back wall.

The projection of nodes and active beams used in intracranial cases and the corresponding shielding load distribution on the surrounding walls are depicted in Fig. 4. The projection was summarized in Table 2. For example, 11 out of 120 intracranial nodes and 13% of intracranial beams projected on the front wall, the “hot spot” of which received 0.13% of the total intracranial MUs. The hot spot only received up to 0.16% of total MU used in the intracranial cases, around 30 times smaller than 5%, the use factor recommended by the guidelines.

**Table 2:** Summation of the projection of intracranial nodes and active beams to the surrounding walls and floor, and the percentage of MU received at the most irradiated area of walls to the total MU used in intracranial treatment.

*Intracranial Patients*	*Front Wall*	*Left Wall*	*Back Wall*	*Right Wall*	*Floor*
Number of Node Projected	11	14	0	4	93
Percent of Beam Projected	13%	9.3%	0	3.3%	74.4%
MU of a Hot Spot / Total MU	0.13%	0.16%	0	0.16%	N/A

#### C.2 Extracranial patient

**Figure 5:**
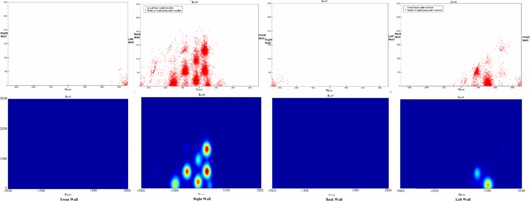
The projection of radiation beams (red dot) and nodes (black dot) used in extracranial cases, and the corresponding MU weighted shielding load distribution.

The projection of node and active beam used in extracranial cases and the corresponding shielding load distribution in the surrounding walls are reported in Fig. 5 and summarized in Table 3. The hot spot received up to 1.01% of total MU used in the extracranial cases, around five times smaller than 5%, the use factor recommended by the guidelines.

**Table 3:** Summation of projection of extracranial nodes and active beams to the surrounding walls and floor, and the percentage of MU received at the most irradiated area of walls to the total MU used in extracranial treatment.

*Extracranial Patients*	*Front Wall*	*Left Wall*	*Back Wall*	*Right Wall*	*Floor*
Number of Node Projected	0	1	0	6	84
Percent of Beam Projected	0.08%	2.1 %	0.07%	6%	91%
MU of Hot Spot / Total MU	0.08%	1.01%	0.06%	8%	N/A

#### C.3 All patients

**Figure 6:**
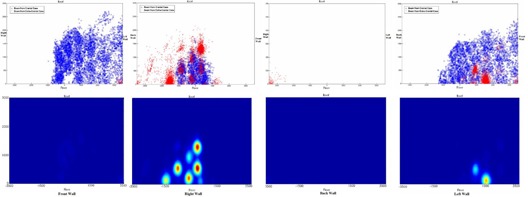
The projection of intracranial radiation beams (blue dot), extracranial beams (red dot), and nodes (black dot), and the corresponding MU‐weighted shielding load distribution.

The distribution of delivered beams for combined intracranial and extracranial patients is displayed in Fig. 6 and summarized at Table 4. Eighty‐five percent of the total projected beams exited through the floor, whereas 76% of the nodes are projected at the floor. Due to mechanical limits of the robot, between 40% and 50% of the total wall area did not receive any direct beams. For the rest of wall we found “hot” spots receiving more than average MUs. The locations of these spots correlated with the projection of the nodes for extracranial treatments. The beam projections on the wall were more spread for intracranial treatments.

**Table 4:** Summation of projection of nodes and active beams to the surrounding walls and floor, and the percentage of MU received at the most irradiated area of walls to the total MU.

*All Patients (Mix of 30% intracranial, 70% extracranial)*	*Front Wall*	*Left Wall*	*Back Wall*	*Right Wall*	*Floor*
Number of Node Projected	11	14	0	4	93
Percent of Beam Projected	3.7%	4.1%	0.05%	6.67%	85.5%
MU of Hot Spot / Total MU	0.07%	0.85%	0.06%	0.98%	N/A

In our practice, with a mix of 29% intracranial and 71% extracranial cases, the hot spot receiving highest intensity of MUs is located at the center of the right wall. The hot spot received no more than 1% of the total MUs, five times smaller than the use factor of 5%, recommended by the guidelines.

The distribution of projected intracranial beams is clearly different from that of extracranial beams. Intracranial beams irradiated a large portion of the front wall, while only a few extracranial beams hit the lower corner of the front wall. For intracranial cases, the number of beams exiting through the left wall is three times the number exiting through the right wall. However, for extracranial cases, the number of beams through the left wall is only 25% of the number exiting through the right wall. No intracranial beams and few extracranial beams exit through the back wall.

**Figure 7:**
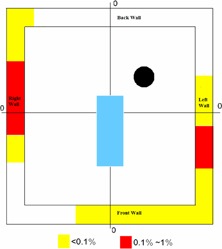
The uneven shielding load distribution of the hypothetical CyberKnife vault. Part of wall (in red) has higher chance (0.1%∼1%) of receiving direct radiation beams than the wall in yellow (<0.1%). The wall in white has not received any direct beam.

In addition, intracranial beams tend to spread further away from the projection of the nodes, while extracranial beams tend to cluster around the nodes, and generate “hot” spots on the wall. Because an extracranial target is usually placed close to the alignment center, the beam's exit point through the target is close to the projection of the node through the alignment center. Consequently, the summation of extracranial beams shows clusters of beams on the wall around the node projection. For intracranial cases, the alignment center is always defined at the geometric center of the skull during the planning process, but an intracranial lesion can be anywhere within the skull. Beams going through the brain lesions can exit at locations that are further from the node's projection through alignment center. Because of this, intracranial beams exit through fairly large regions on the wall, but with very low density, due to the spread (Fig. 7).

## IV. DISCUSSION & CONCLUSIONS

Because the use factor is small and the modulation factor is large, the leakage barrier requirements for a CyberKnife are comparable to primary barrier requirements, or maybe even larger in certain directions. We identified a large modulation factor (7.43) in our clinic, but this was smaller than the modulation factors recommended in the guidelines. It has resulted from our clinical preference and planning strategy, which emphasizes the dose falloff over dose conformality in radiosurgery. We normally choose a collimator size that is slightly smaller than the smallest dimension of PTV, which does not compromise dose gradient and will have a smaller modulation factor. Particularly, we prefer to use isocentric plans to treat small tumors with a regular shape. The isocentric plans generally have lower modulation factor than nonisocentric plans which require more modulation to generate a conformal dose cloud. Iris dynamic collimator has been used in many clinics to allow the planner to choose multiple collimator sizes to treat a tumor, which improves the planning quality and allows for a similar treatment delivery time with the single collimator plan. Multiple studies have reported 25%∼50% of MU reductions using Iris comparing with single‐cone collimator plan.^7^, ^8^, ^9^ However, based on our large cone selection in planning, we do not think the utilizations of Iris would affect the modulation factor in this study (7.43).

We found our maximum use factor to be around 1%, much less than the 5% recommended in the guidelines. The fivefold difference arose from different estimating methods. Guidelines estimated the use factor as the ratio of the largest number of MUs from a node divided by the total number of MUs used in one clinical plan. The ratio was estimated for each of 55 plans and 5% was found as the average plus one standard deviation. This method did not consider the fact that the targets of different plans are at different locations leading to the beams from a node to be projected at different locations in different plans. CyberKnife beams do not shoot though the same target position, which is different with the pattern from gantry‐mounted linacs, where all beams pass through the same isocenter. We recommend using a 5% for the sometimes‐required ‘in‐any‐one‐hour’ exposure estimation because that value was derived from a particular real plan. The typical delivery time is also 1 hr for most of treatments.

Our study reproduced the composite dose distribution on the shielding wall from the 1370 clinical plans, and estimated the use factor based on the composite distribution. Since the composite data were generated based on every active beam's specific source and target position, the effect of various target positions in different cases was taken into account in the shielding load distribution. In other words, because the targets change position between patients while node position remain unchanged, beam projection from a single node can result in different contact point on the wall between different patients and this fact was included in our study but not in guidelines. The spread of beam projecting locations smear down the composite use factor to 1%. The spread factor is around 1/5 and can be as low as 1/30 in the intracranial cases. The resulted 1% use factor is more appropriate for the integral dose estimation.

With the small use factor and the small field size of the beam, the overall workload for a given barrier wall to be shielded in any one direction is small. This can result in a thin primary barrier that meets the regulatory limit for integral dose in the shielding design. However, the instantaneous dose rate can be considerably higher than the customary limit,^(10)^ and “result in an unacceptable ‘in‐any‐one‐hour’ dose equivalent inside beam exit areas”,^(2)^ which can be a concern for meeting regulatory limits for uncontrolled areas. As a result, we do not suggest designing the primary barrier using a use factor of 1%, at least to uncontrolled area. However, it is beneficial to know that the real integral dose may be five times lower when 5% is adopted as user factor.

The vendor recently released a new model CyberKnife (M6) with a different robot position. For this new unit, the shielding load distribution may differ from we have experienced over the past five‐year period. It may, therefore, be wise to continue to make the surrounding walls all primary barriers, if possible, to accommodate the new configuration of the M6 CyberKnife or a future model CyberKnife. However, for the G4 model, we suggest that the uneven primary shielding load might be considered in the design of shielding requirement and in the design of the facility layout. For example, for a conventional linac vault that is modified to accommodate a G4 CyberKnife, it may not be necessary to add additional shielding around the whole vault but only to the area that may be directly irradiated. For a new vault with uniform thickness surrounding shielding (due to the ease of construction), the facility layout might be designed to put lower occupancy rooms in the area of higher frequency of direct irradiation. In particular, it may be wise to place the door in an area where no direct primary beams project, so that the door is only a secondary barrier, which would avoid direct irradiation to the gap between door and door frame. Because the shielding load pattern is different between intracranial case and extracranial cases, the patient mix can also be a factor to be considered in shielding design, particularly for the CyberKnife programs intended for exclusively intracranial cases or exclusively extracranical cases.

## ACKNOWLEDGMENTS

The authors are grateful to David Schaal, PhD, for his editorial assistance on this paper.

## Supporting information

Supplementary Material

Supplementary Material
