# Supplementary material for: Radiation shielding evaluation based on five years of data from a busy CyberKnife center
Source: J Appl Clin Med Phys. 2014 Nov 8;15(6):313–22. doi: 10.1120/jacmp.v15i6.4575 (PMC5711125; doi:10.1120/jacmp.v15i6.4575)
Supplement: Supplementary file 2 — Supplementary Material [file ACM2-15-313-s002.doc]

**Radiation Shielding Evaluation Based on Five Years of Data from a Busy CyberKnife Center**

**Introduction:** We examined the adequacy of existing shielding guidelines using 5-year clinical data from a busy CyberKnife Center.

**Methods:** From June 2006 through July 2011, 1,370 patients were treated with a total of 4,900 fractions and 680,691 radiation beams using a G4 Cyberknife. Prescription dose and total Monitor Units (MU) were analyzed to estimate the shielding workload and modulation factor. In addition, based on the beam’s radiation source position, targeting position, MU and beam collimator size, the Matlab program was used to project each beam toward the shielding barrier. The summation of the projections evaluates the distribution of the shielding load.

**Results:** On average, each patient received 3.6 fractions, with an average 9.1 Gy per fraction prescribed at the 71.1% isodose line, using 133.7 beams and 6,200 MU. Intracranial patients received an average of 2.7 fractions, with 8.6 Gy per fraction prescribed at the 71.4% isodose line, using 133 beams and 5,083 MU. Extracranial patients received an average of 3.94 fractions, with 9.2 Gy per fraction prescribed at the 71% isodose line, using 134 beams and 6,514 MU. Most used collimator sizes for intracranial patients were usually smaller (7.5 to 20 mm) than for extracranial patients (20 to 40 mm). Eighty-five percent of the beams exited through the floor. roboanicbout of the surrounding wall area received no direct beams. For the ret of the wall, we found “hot” spots areas tceived above-average MU. The distribution these ares spots was were correlateh distribution projection es for extracral treatments. In comparison, , and thus there Tthe beamsroionshe wae morespreaded ere fewer hot spots for intracranial treaeany spot area received from intracranial treatmen is less than 0.25% of total MU used for intracranl treatments, and received from extracranial treatment and is less than 1.2% of total MU used for extraal treatments. The combination of workload, modulation factor and use factor in our practice are about 10-fold less than recommendations in the existing CyberKnife shielding guidelines.

**Conclusion:** The current guidelines were found to be adequate for shielding, even in a busy center. There may be a potential to reduce shielding in areas with no or few direct beams in the current G4 model. . The uneven distribution of use factor we have found may be considered in the vault design if the newer unit treats with similar plan.As a newer-model CyberKnife (M6) has recently been introduced, the patterns of usage reported here may be changed in the future.

**I. INTRODUCTION**

Unlike gantry-based linear accelerators (linacs), which deliver radiation within a single plane through the isocenter, the CyberKnife delivers many 6-MV non-coplanar pencil beams from a wide solid angle around the target. Every beam originates from one of 120 pre-configured positions, or “treatment nodes”, that are nearly evenly distributed around the imaging alignment center. The centroid of a treatment target is located close to the alignment center but not necessarily right on the alignment center. More than one beam can be directed from a single node (aiming at different portions of the target) and some evdes necmay not havetive beam depending upon planning optimization. Because of this configuration, CyberKnife beams exit to a very large part of the surrounding walls. As a result, CyberKnife vaults require a much larger primary shielding barrier than Linacs, but the use factor for each beam is much smaller. Another difference from linac treatments is that the CyberKnife is used almost exclusively for radiosurgery and stereotactic body radiotherapy (SBRT), so a very high per-fraction dose is delivered in up to 5relatively ctions per treatment course.

These different characteristics have been addressed in the available CyberKnife shielding guidelines. Three of these guidelines are widely accepted by CyberKnife physicists: NCRP 151 has been the gold standard guideline for shielding design^(1)^. A book chapter published in 2005 ^(2)^, “CyberKnife Treatment Room Design & Radiation Protection”, was the first published guideline and stated well the shielding concepts that still stand today. These two guidelines estimated the primary workload and use factor based on the experience of the Georgetown University CyberKnife program ^(3)^. The shielding white paper ^(4)^ from Accuray Incorporated, manufacturer of the CyberKnife, is valuable in addressing leakage dose and addressing secondary barrier requirements, based on multiple centers’ clinical data on modulation factor. All three of these guidelines propose that all surrounding walls serve as the primary shielding barrier with 5% as a uniform use factor for each wall.

We examined the adequacy of these shielding guidelines using 5 years of clinical data from a busy CyberKnife program. We report our workload and shielding-related clinical characteristics and provide additional suggestions based on our finding of an uneven distribution of use factor across the vault floor and walls.

**II. METHODS**

II.A. Data collection

We treated 1,370 patients, including 29% (392) intracranial cases and 71% (978) extracranial cases, with a total of 4,900 fractions at Philadelphia Cyberknife from June 2006 through July 2011. The Cyberknife mMdel wa atG4ith the software versin 7.x, upgraded to version 8.x in February 2009. Every patient was treated using one or two cone collimators selected from 12 circular field sizes: 5, 7.5, 10, 12.5, 15, 20, 25, 30, 35, 40, 50 and 60 mm in diameter. A oOne-hed path with 120 nodes was used to treat all brain patients except for 11 trigeminal neuralgia patients treated with a special trigeminal path. The Oone-bdy path has around 90 nodes, which excludes the nodes distributed on the apex of the skull due to the patient safety concerns and the robot mechanical limitations. Theone-body path was adopted to treat all extracranial patients, except for the prostate patients who were treated using a special prostate path.

Clinical data including the prescription dose, fractionation schemes and MUs were retrospectively analyzed to estimate the workload and modulation factor. The 680,691 delivered radiation beams were simulated with usatlab software to estimate the use factor. Based on the geometric positions of each radiation sources and its secific targeting position, and weighted by monitor units (MU) and collimator size, each beam was projected to the floor or the surrounding barrier wall. (Fig. 1). Different with Linac technology whose beams from the same gantry angle go through the same isocenter and project to the same directions, the Cyberknife beams from a node do not project to the same directions for the two following reasons: 1) within one Be aware thalan, the beams from a node will irradiate different potions of the target with partialsome or none overlap with otherr. 2The targets in the differet plans usually arelocate at different relative ptions relative to the alignment center. In, the target can be at various loc

The summation of the projected MUs evaluates the distribution of the shielding load in a hypothetical vault with the inner space of 7m x 7m x 3m. (Fig.ure 21). In most cases the CyberKnife robots are positioned on the left superior side of couch, but in our center the robot is on the right superior side of the couch (known as the mirrored position). The distance from the radiation source to the entry point of a shielding wall is not factored in the summation to estimate the use factor, since the distance is an independent factor in shielding design. To simplify the calculation, “perfect” beams with 100% uniform dose in the field and 0% dose outside the field (as distinct from the real beam and its penumbra), were was used.


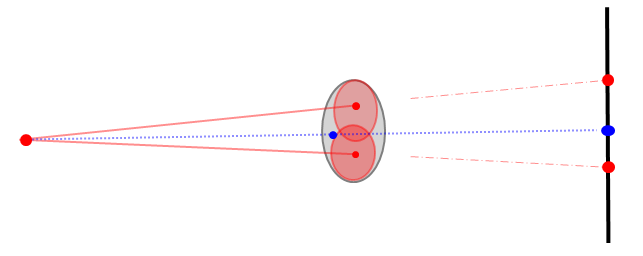


Fig.1: The illustration of beam projection and node projection. Assume there are two beams (with central axis marked as solid red line) from one treatment node (red solid dot on left), each irradiating a different portion of the target (in gray). The beams are projected to the wall on the right and the node projected through the alignment center (solid blue dot in the tumor) to the different location of the wall.


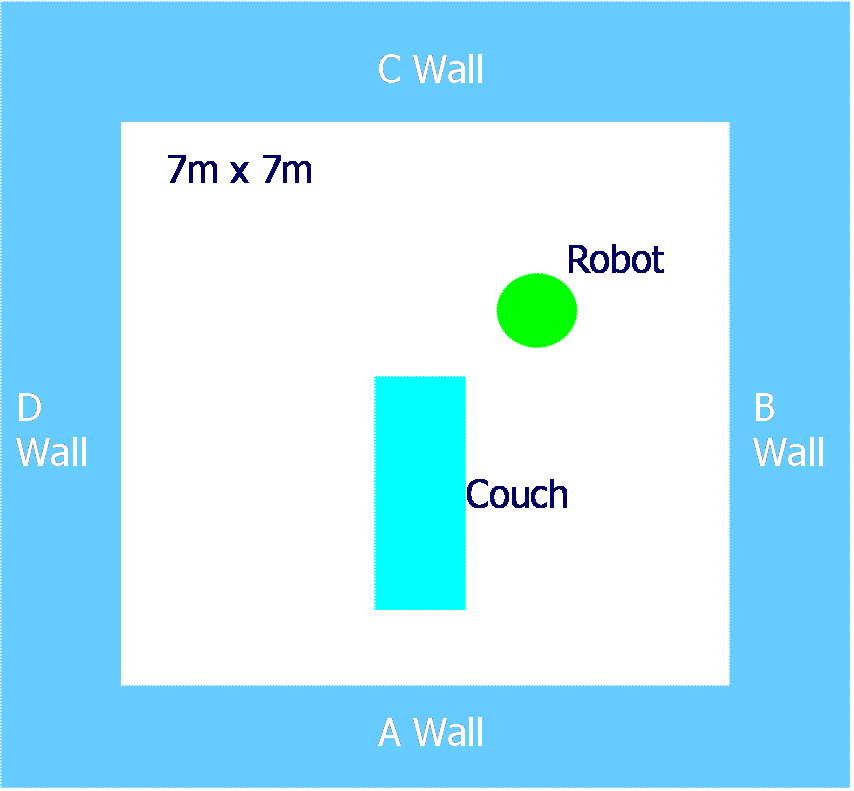


Fig. 2: The layout of a hypothetical square Cyberknife vault with four surrounding walls, marked as A, B, C and D wall.

: Screen scale issue. Will fix it. II.B. Shielding guidelines

Because of the fact that CyberKnife’s primary beams exit onto a large portion of the surrounding walls and the potential of relocating the robot in the newly designed Cyberknife model (M6), all three shielding guidelines recommend designing the surrounding walls as primary barriers and designing the roof or ceiling as a secondary barrier because a CyberKnife is limited in its ability to point in an upward direction. .

Based on primarily on the Georgetown experience, which found the largest number of beams shooting from one node is about 5% of total number of beams in a single plan, a use factor of 5% is recommended. Georgetown’s experience also found an average modulation factor of 15 for both intracranial and extracranial cases. A survey of multiple CyberKnife centers, cited in Accuray’s shielding white paper, found an average modulation factor of 12.5 (range from 9.2 to 25) and also recommended 15 as modulation factor as a reasonable conservative commendation. BecceSthe small field used inproduf a Cye relaiey small comparing with the Linac, the guidelines suggest that the amount of radiation wolrdkload generated from ptent scatter is negligible.

We summarize the guidelines recommended parameters in Table 1, along with our clinical data.

|  | Guidelines Recommendation | | | Our Clinical Data |
| --- | --- | --- | --- | --- |
|  | NCRP 151 | White Paper  (ref 4) | Book Chapter  (ref 2) |
| Max Num. of Tx per Day | 8 | 8 | 6 | 9 |
| Average Dose Per Tx (Gy) | 12.5 | 12.5 | 12.5 | 9.1 |
| Workload (Gy/week at 80cm SAD) | 500 | 500 | 375 | 400.4 |
| MU per Tx |  | 16,000 |  | 6200 |
| Modulation Factor | 15 | 15 | 15 | 7.43 |
| Use Factor | 0.05 | 0.05 | 0.05 | 0.01 |

Table 1: Summary of recommended parameters in the Cyberknife shielding design guidelines and the parameters found from the clinical data of Philadelphia Cyberknife

**III. RESULT**

III.A Primary Shielding Load

At PhillyCyberknife, patients on average received 3.6 fractions, with a mean 9.1 Gy per fraction prescribed to the 71.1% isodose line. Most frequently used fractionation schemes were 1 (14%), 3 (33.8%) and 5 (41.2%) fractions. Specifically, the intracranial patients received an average of 2.7 fractions, with 8.6 Gy per fraction prescribed at the 71.4% isodose line. 32.5%, 22.8% and 37% of intracranial patients received 1, 3 and 5 fractions, respectively and a few intracranial patients received 2 or 4 fractions. The extracranial patients received an average of 3.94 fractions, with 9.2 Gy per fraction prescribed at 71% isodose line. 40.1%, 8.7% and 46% of extracranial patients received 3, 4 and 5 fractions respectively and only Few extracranial patients received 1 or 2 fractions.

There have been a couple of studies supporting faster dose fall-off when performing SBRT along with higher dose heterogeneity(^5,7^) TIn our practice the averae prescription isodose has dropped gradually, from the high 70% range to the high 60% range during the last 5 years (Figure 23.a.1is corresponds t a tend in our clinical emphasis practice toward madose fall-off in surrounding tissue, and allowing higher greater heterogeneitn treatment volumes. The exception is for the prostate patients where maintained the prescribed isodose line at around 83%.

On average, 27 patients (*SD* 7) were treated with 95 fractions (*SD* 25) every month. During the highest-volume year, 347 patients were treated with 1,203 fractions. In the highest-volume month, 41 patients were treated with 145 fractions. During the highest-volume week, 44 fractions were delivered to a total of 381 Gy. The history of our monthly patient treatment volume is reported in Fig.3.b.
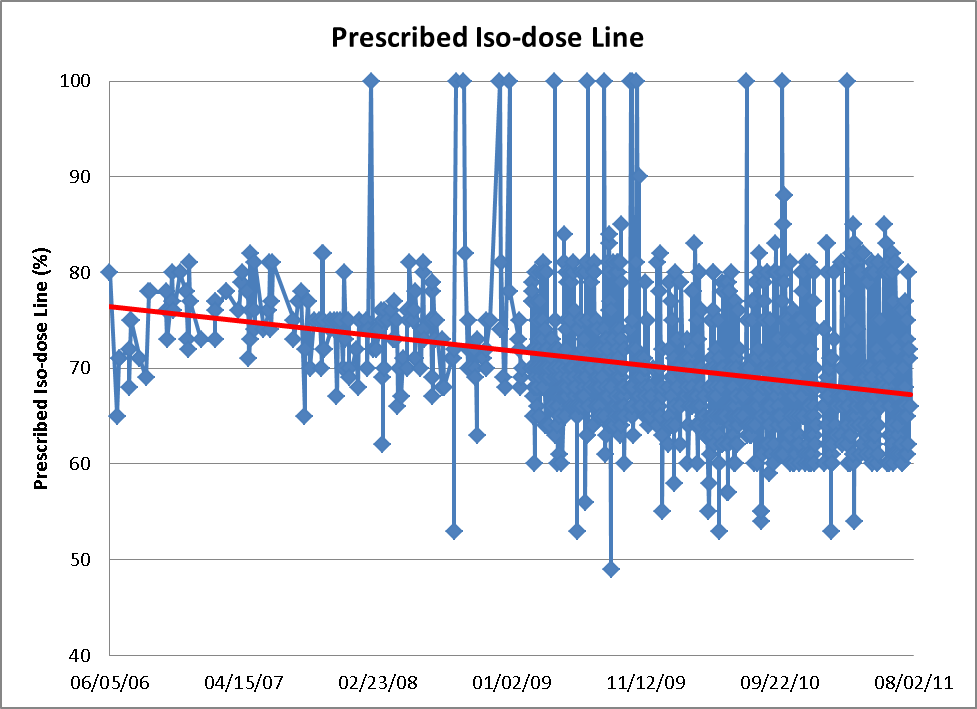


Fig. 3. The history of prescribed isodose line (left 3.a) and the number of patients treated per month (right 3.b)

The chance of using 5, 7.5, 10, 12.5, 15, 20, 25, 30, 35, 40, 50 and 60 mm collimator are 1.1%, 4.3%, 5.3%, 5.5%, 8.5%, 14.8%, 11.2%, 13.4, 14.9%, 12.7% and 5.6%. Most commonly used collimator sizes for intracranial patients were usually smaller (7.5, 10, 12.5, 15 and 20 mm) than extracranial patients (20, 25, 30, 35 and 40 mm).

III. B Leakage Load and Modulation Factor

On average, intracranial patients received 8.61 Gy per fraction with 5,083 MUs (*SD* 2268) and 133 (*SD* 50) beams, which resulted in a modulation factor of 6.2 (5083 MUs /861 cGy) for intracranial patients. A mean of 38.2 MUs per beam per fraction were used in intracranial treatments. With an average 6517 (*SD* 3040) MU delivered to extracranial patients, who receives 9.2 Gy per fraction in 134 (*SD* 49) beams*SD*9)e modulation factor for extracranial patients was 7.93, higher than intracranial patients due to greater tissue attenuation. A mean of 53.8 MUs per beam per fraction was delivered in these extracranial treatments. The combined average modulation factor was 7.43, which is about 50% of the recommended value or the multi-site survey value seen in the published fromdelines. As noted, our practice has been emphasizing dose fall-off in the surrounding tissue, and conformality index is not our first planning optimization goal, since the an with higher conformality often have hslower dose fall-off and requires a lot more MUs.

III. C Shielding load distribution and use factor

III.C.1 Intracranial Patient

The projection of nodes and active beams used in intracranial cases and the corresponding shielding load distribution on the surrounding walls are depicted in Fig. 4. The projection is summarized in Table 2. For example, eleven out of 120 intracranial nodes and 13% of intracranial beams projected on the A wall, the highest-load portion of which received 0.13% of the total intracranial MUs. soe flo

| Intracranial Patients | Wall A | Wall B | Wall C | Wall D | Floor |
| --- | --- | --- | --- | --- | --- |
| Number of Node Projected | 11 | 14 | 0 | 4 | 93 |
| Percent of Beam Projected | 13% | 9.3% | 0 | 3.3% | 74.4% |
| MU of Hot Spot / Total MU | 0.13% | 0.16% | 0 | 0.16% | N/A |

Table 2: Summation of the projection of intracranial nodes and active beams to the surrounding walls and floor, and the percentage of MU received at the most irradiated area of walls to the total MU used in intracranial treatment.


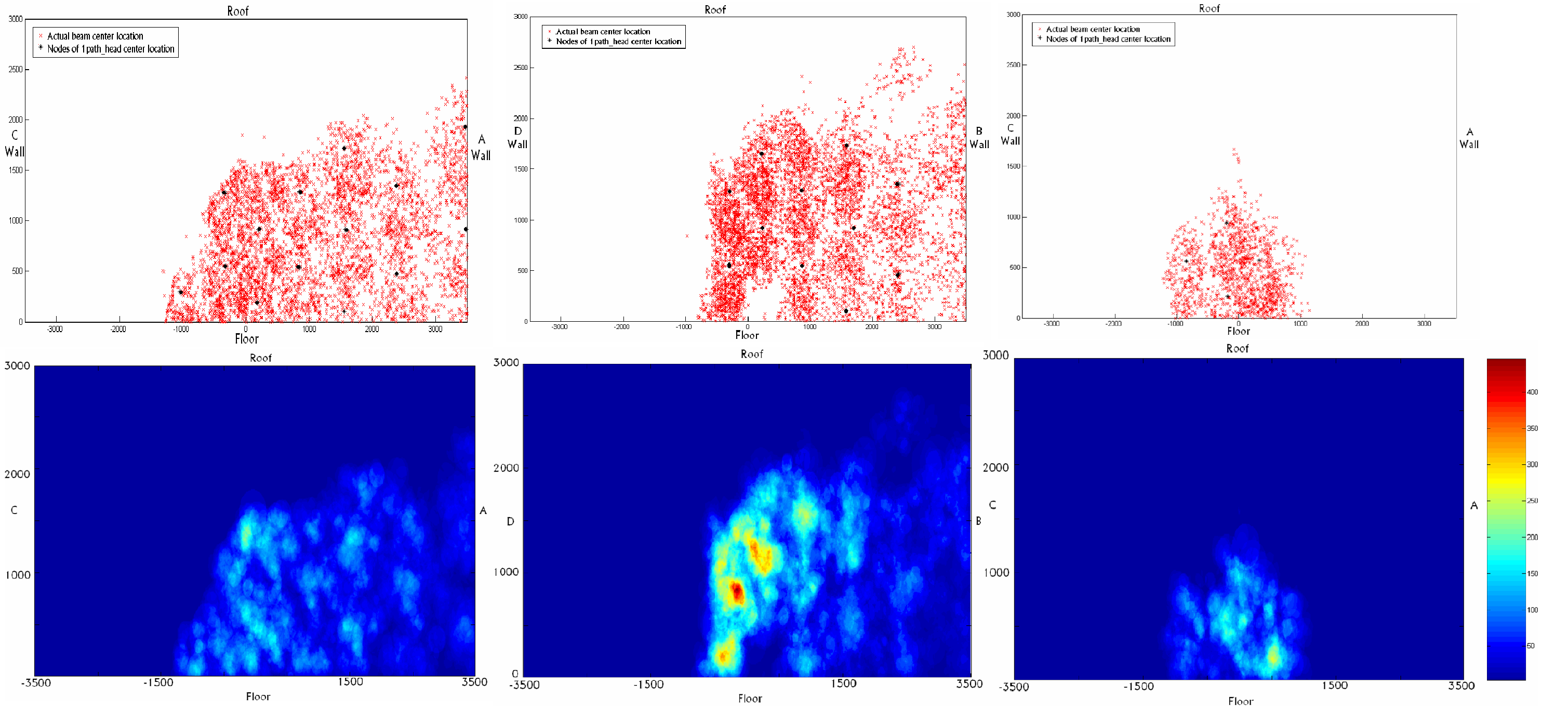


Fig.4 . The projection of radiation beams (red dot) and nodes (black dot) used in intracranial cases, and the corresponding MU weighted shielding load distribution on A wall (left), B wall (middle) and D wall (Right). C wall received no active beam.

III.C.2 Extracranial Patient

The projection of node and active beam used in extracranial cases and the corresponding shielding load distribution in the surrounding walls are reported in Fig.5 and summarized at Table 3.4. Zll A. Only 0.08% of the extracranial beams projectedt rd o lf the A wall,ghest l8% of the total extracranial MUsone nodeived 6 nodes and 8 % of the beams, with the highest load area getting 1.2% of the total extracranial MUs. The rest of the nodes and beams (around 90%) projected toward at the floor.

| Extracranial Patients | Wall A | Wall B | Wall C | Wall D | Floor |
| --- | --- | --- | --- | --- | --- |
| Number of Node Projected | 0 | 1 | 0 | 6 | 84 |
| Percent of Beam Projected | 0.08% | 2.1 % | 0.07% | 6% | 91% |
| MU of Hot Spot / Total MU | 0.08% | 1.01% | 0.06% | 8% | N/A |

Table 3: Summation of projection of extracranial nodes and active beams to the surrounding walls and floor, and the percentage of MU received at the most irradiated area of walls to the total MU used in extracranial treatment.


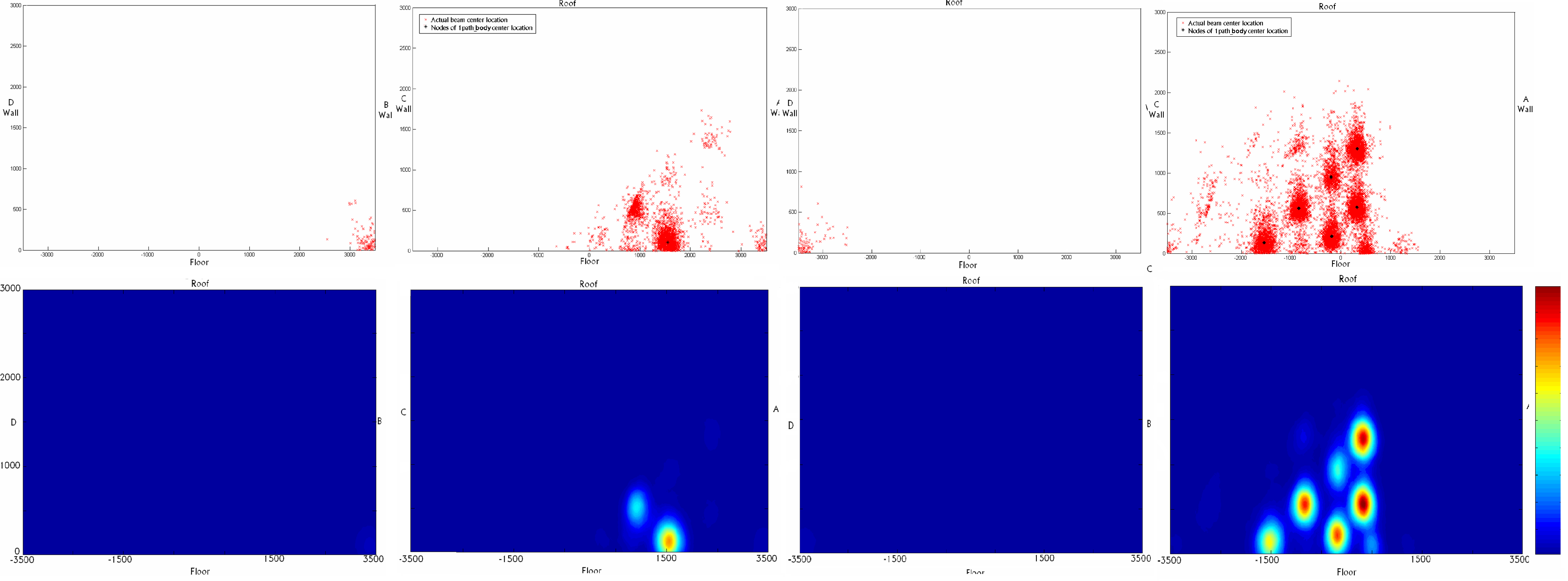


Fig. 5. The projection of radiation beams (red dot) and nodes (black dot) used in extracranial cases, and the corresponding MU weighted shielding load distribution on A wall, B wall, C wall and D wall (from left to right).

III.C.3: All Patients

The distribution of delivered beams for combined intracranial and extracranial patients is displayed in Fig. 6 and summarized at Table 4. Eighty-five percent of the total beams exited through the floor. Due to mechanical limits of the robot, between 40 to 50% of the total wall area did not receive any direct beams. For the rest of wall we found “hot” spots receiving more than average MUs. The locations of these spots correlated with the projection of the nodes for extracranial treatments. The beam projections on the wall were more spread for intracranial treatments.

spot received wasith a mix of 29% intracranial and 71% intracranial cases, the hot spot receiving highest intensity of MUs is located at the center of the D wall. And it receives is no more than 1f the total MUs.

| All Patients (Mix of 30% intracranial, 70% extracranial) | Wall A | Wall B | Wall C | Wall D | Floor |
| --- | --- | --- | --- | --- | --- |
| Number of Node Projected | 11 | 14 | 0 | 4 | 93 |
| Percent of Beam Projected | 3.7% | 4.1% | 0.05% | 6.67% | 85.5% |
| MU of Hot Spot / Total MU | 0.07% | 0.85% | 0.06% | 0.98% | N/A |

Table 4: Summation of projection of nodes and active beams to the surrounding walls and floor, and the percentage of MU received at the most irradiated area of walls to the total MU.


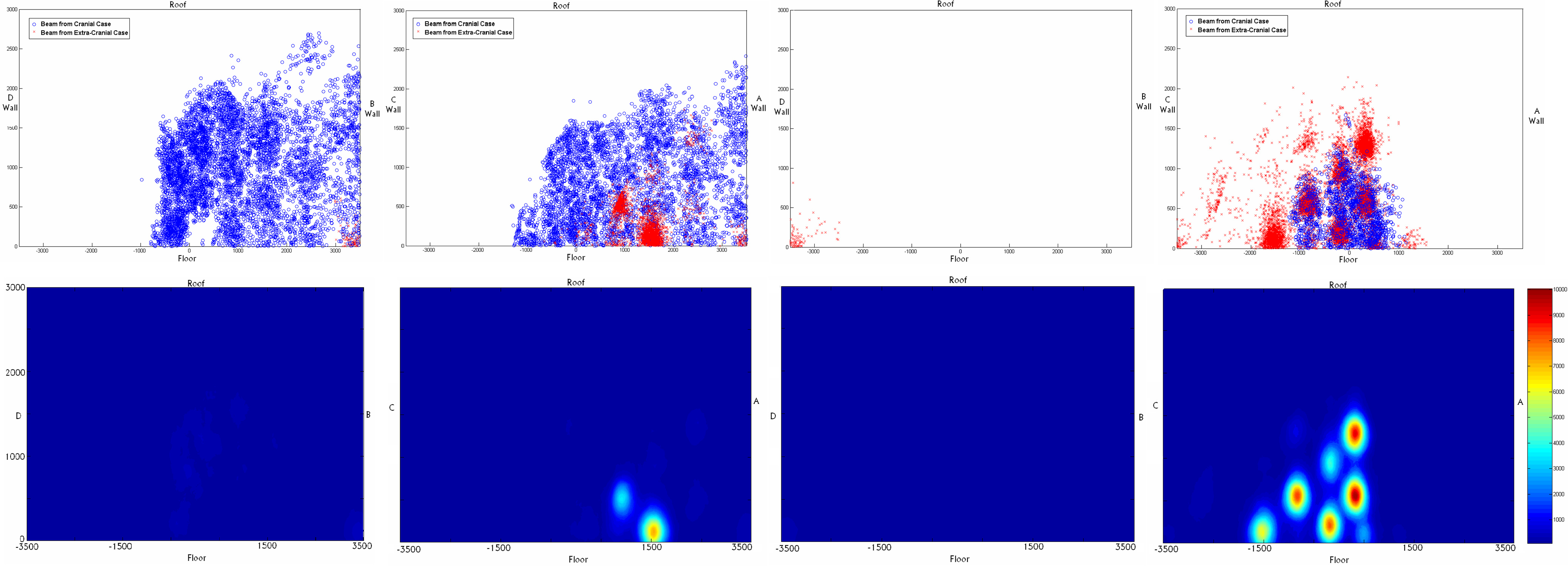


Fig. 6. The projection of intracranial radiation beams (blue dot), extracranial beams (red dot) and nodes (black dot) and the corresponding MU weighted shielding load distribution on A wall, B wall, C wall and D wall (from left to right).

delidistribution of intracranial beams is clearly different from that of extracranial beams. Intracranial beams irradiated a large portion of the A wall, while only a few extracranial beams hit the lower corner of the A wall. For intracranial cases, the number of beams exiting through the B wall is three times the number exiting through the D wall. However, for extracranial cases, the number of beams through the B wall is only 25% of the number exiting through the D wall. No intracranial beams and few extracranial beams exit through the C wall.

usuIn addition, intracranial beams tend to spread further away from the projection of the nodes, while extracranial beams tend to cluster around the nodes, and generate “hot” spots on the wall. Because extracranial target is usually placed close to the imaging center, the beam's exit point through the target is close to the projection of the node through the imaging center. Consequently, the summation of extracranial beams shows clusters of beams on the wall around the node projection. For intracranial cases, the imaging center is always defined at the geometric center of the skull during the planning process, but an intracranial lesion can be anywhere within the skull. Beams going through the brain lesions can exit at locations that are further from the nodes' projection through image center. Because of this, intracranial beams exit through fairly large regions on the wall, but with very low density, due to the spread.

Fig. 7 summarizes the uneven distribution of the shielding load on the surrounding walls. About 40% of the wall (in white) received no direct beams during the 5 years studied, and approximately 40% of total wall (in yellow) received less than 0.1% of the direct beams. The remaining 20% of the total wall space (in red) receives between 0.1% and 1% of the direct beams.


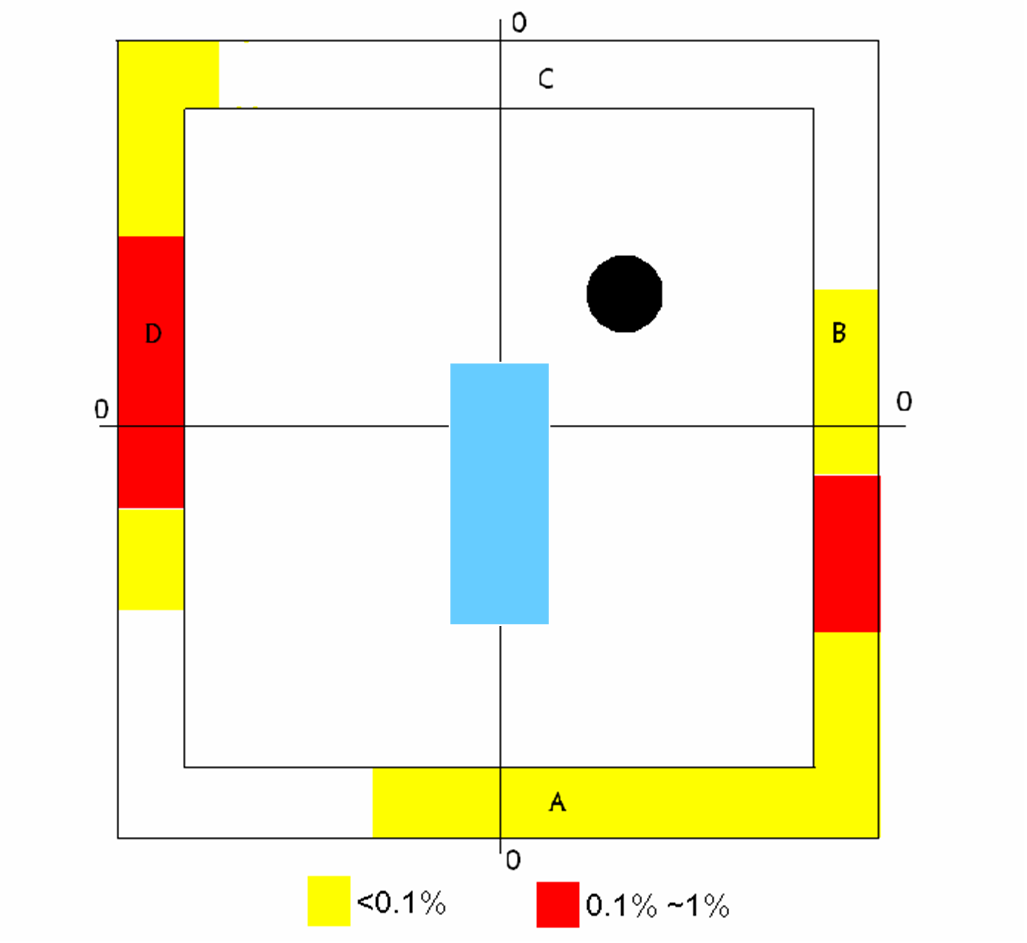


Fig. 7: The relative uneven shielding load distribution of the hypothetical Cyberknife vault. Part of wall (in red) has higher chance (0.1% ~ 1%) receiving direct radiation beams than the wall in yellow (< 0.1%). The wall in white has not received any direct beam in this study. To authors: again I don’t see why this is not a square since the dimensions of length and width are identical.

**IV. DISCUSSION**

Because the use factor is small and the modulation factor is large, the leakage barrier requirements for a CyberKnife are comparable to primary barrier requirements, or may be even larger in certain directions. We identified a large modulation factor (7.43) in our clinic, but this was smaller than the modulation factors recommended in the guidelines. It has resulted from our clinical preference and planning strategy, which emphasizes the dose fall-off over dose conformality in radiosurgery. We normally choose a collimator that is slightly smaller than the smallest dimension of PTV, which does not compromise dose gradient and will have smaller modulation factor. Particularly, we tend to use isocentric plans to treat small or regular shaped tumor, which results in a lower modulation factor than non-isocentric plans which require more modulation to generate a conformal dose cloud.

aroundtudy, Because tseren, the mohe what you are saying here. The senteWe found our maximum use factor to be around 1%, much less than the 5% recommended in the guidelines. The 5 fold difference arose from different estimating methods. Guidelines estimated the use factor as the ratio of the largest number of MUs (or beams) from a node divided by the total number of MUs (or beams) used in one clinical plan. The ratio was estimated for each of 20 plans and 5% was found as the average. This method did not consider the fact that the targets of different plans are at different locations, as such, the beams from a node projected at different locations in different plans. The fact that Cybernife beams do not shoot though the same target position is different with Linac, where the Lianc beams always shoot through the same isocenter. In our opinion,We recommend using 5% is more appropriate for ‘in-any-one-hour’ estimation since it is derived from individual plan, and it usually takes one hour to deliver a plan.

Our study re-produced the composite dose distribution on the shielding wall from the multiple plans first, and estimated the use factor based on the composite distribution. Since the composite data was generated based on every active beam’s source and target position, the effect of various target positions at different plans was took into account in the distribution. The spread of projected beam positions smear down the composite use factor to 1%. The 1% use factor is more appropriate for integrate dose estimation.

With the small use factor and the small field size of the beam, the overall workload for a given barrier wall to be shielded in any one direction is small. This can result in a thin primary barrier that meets the regulatory limit for integral dose in the shielding design. However, the instantaneous dose rate can be considerably higher than the customary limit (7), and “result in an unacceptable ‘in-any-one-hour’ dose equivalent inside beam exit areas” (2), which can be a concern for meeting regulatory limits for uncontrolled areas. As a result, we do not suggest designing the primary barrier using a use factor of 1%, at least to uncontrolled area. However, it is beneficial to know that the real integrate dose will be lower when 5% is adopted as user factor.

beammeasor of

The vendor recently released a new model Cyberknife (M6) with a different robot position.,aor this new unit the shielding load distribution wiy possibly be different than we have encountered over the past five-year period. It may therefore be wise to continue to make the surrounding walls all primary barriers, if possible, to accommodate the potential new configuration of the M6 CyberKnife or the future Cyberknife. However, to the G4 model, we suggest that the uneven primary shielding load inof the4 dee considein the shielding design of requirement,, in terms of shielding calcufacility layout once practicepatient referral pattere mortablr exaLiaccommodate a G4 CyberKie, it may not be necessary to add additional shielding around the whole room valult but only to the area that may be directly irred With theFor a new vault of evenwith uniform-thickness sur shielding (due easeiness of construction), the facility layout might can be ned to put lower occupancy roomsess occupied e in the area of higher frequency of dir particular, Particularly, it may be wise to place the door in an area t primary beams project, so that the door is only a secondary barrier, which would avoid direct irradiation to the gap between door and door frame. In addition,Because the shielding load pattern is differentracraal case and extracranial cases., tThe patient mix can also be a factor to be considered i shelding design, particularly for the CyberKnife programs intended for exclusively intracranial cases or exclusively extracranical cases exclusively.only.

**Acknowledgments**

Redacted to preserve anonymity of review process.

References

1. NCRP Report No. 151, Structural Shielding Design and Evaluation for Megavoltage X- and Gamma-Ray Radiotherapy Facilities. Chapter 7.2 Robotic Arm Stereotactic –Radiosurgery room P 148~157

2. James Rodgers, CyberKnife Treatment Room Design and Radiation Protection, Chapter 5, Robotic Radiosurgery, Vol.1, CyberKnife Society Press, Sunnyvale, CA, (2005)

3. James E. Rodgers, Analysis of tenth-value-layers for common shielding materials for a robotically mounted stereotactic radiosurgery, H. Phys. 92, 379-386, (April 2007)

4. Anuj K. Purwar etc. Tenth-Value layer Measurements of leakage radiation and secondary Barrier shielding calculations for the Cyberknife robotic radiosurgery system. Accuray White paper 2009

5. 6. Alexander Martin etc. [Choosing a Value for the Prescription Isodose: How this Affects the External Dose Gradient in CyberKnife Planning of Intracranial Lesions](http://therss.org/document/docdownload.aspx?docid=840). Cyberknife summit (2011)

7.

6. Jun Yang. The dosimetry of Cyberknife radiosurgery. Radiosurgery Society meeting (2012)

7. Jun Yang etc. An Evaluation of the Use Factor in CyberKnife Shielding Design, 2007 AAPM Annual Meeting
